# Metabolomic Analysis of Maize Response to Northern Corn Leaf Blight

**DOI:** 10.3390/metabo15020113

**Published:** 2025-02-10

**Authors:** Yingnan Gu, Bowei Yan, Ye Yang, Ying Huang, Xin Liu, Shubin Liu

**Affiliations:** 1 Institude of Agricultural Remote Sensing and Information, Heilongjiang Academy of Agricultural Science, Postdoctoral Workstation of Heilongjiang Academy of Agriculture and Science, No. 368, Xuefu Road, Nangang District, Harbin 150086, China; guyingnan@haas.cn (Y.G.); yangye202412@163.com (Y.Y.); huangying1011@126.com (Y.H.); 2 Institute of Industrial Crops, Heilongjiang Academy of Agricultural Science, Harbin 150086, China; yanbowei21@outlook.com; 3 Heilongjiang Academy of Black Soil Conservation and Utilization, Heilongjiang Academy of Agricultural Science, Harbin 150086, China

**Keywords:** *Zea mays* L., northern corn leaf blight, metabolomic, metabolic pathways

## Abstract

**Background:** As a major food crop, maize is highly susceptible to pathogenic bacteria, which greatly reduces its yield and quality. Metabolomics reveals physiological and biochemical changes in organisms and aids in analyzing metabolic changes caused by various factors. **Methods:** This study utilized metabolomics to examine maize’s metabolic changes after NCLB infestation, aiming to uncover related pathways and potential biomarkers. The metabolite measurements were performed during the maize silking stage. **Results:** PCA showed an obvious dispersion between the treated and untreated groups. OPLS-DA identified 1274 differential metabolites, with 242 being downregulated (mainly phenolics and esters) and 1032 upregulated (primarily organic acids, amino acids, sugars, and derivatives). KEGG annotation revealed 50 affected metabolic pathways, and the biosynthesis of secondary metab-olites and amino acids was significantly enriched. **Conclusions:** We hypothesized that metabolic pathways related to sugar metabolism, proline metabolism, and jasmonic acid synthesis are associated with NCLB susceptibility. These findings provide critical insights into the metabolic responses of maize to biotic stress, offering a theoretical basis for future research on plant resistance mechanisms.

## 1. Introduction

Maize (*Zea mays* L.) is one of the most important food crops and cash crops in China. Outbreaks of maize diseases have seriously affected China’s food security and national economic development [1]. NCLB, a major foliar disease, is prevalent in all maize-producing regions worldwide. It can reduce yields by up to 20% in typical outbreaks and over 50% in severe cases [2]. After maize is infected with the disease, its metabolism is significantly altered, resulting in changes in physiological and biochemical parameters, and the content of characteristic metabolites is also different. These metabolic changes allow for an early prediction and assessment of maize big blotch disease progression [3]. Metabolomic analysis to identify these metabolites is crucial for forecasting disease outbreaks, improving control strategies, ensuring food security, lowering production costs, boosting agricultural efficiency, and fostering sustainable development.

NCLB, caused by *Setosphaeria turcica*, is a destructive leaf pathogen affecting maize at various growth stages [4]. It is primarily transmitted via air currents, rainwater, and seeds. Upon inoculation with the pathogen, its conidia germinate under suitable conditions. The germinating tubes penetrate the leaves, allowing the pathogen to spread between cells, resulting in small chlorotic lesions. Over time, these lesions expand into spindle-shaped large lesions, severely impairing photosynthesis and reducing yields. During the growing period of corn, medium-temperature, high-humidity, and low-light climate conditions are conducive to the occurrence and prevalence of big spot diseases. Favorable conditions include moderate temperatures, high humidity, and low light, which promote disease spread from lower to upper leaves, potentially killing plants. The pathogen overwinters in maize residue as mycelium or conidia, which can spread long distances via wind. In general, the yield is reduced by approximately 20%, and in severe cases, by 50% [5]. Therefore, this disease remains a major constraint in maize production worldwide.

After plant leaves are infested with *Phytophthora infestans*, the pathogen takes up the nutrients and water necessary for reproduction and expansion from the host. This causes significant changes in its metabolism, resulting in changes in leaf color, morphology, structure, and physiological and biochemical parameters, including changes in its characteristic metabolites, membrane system, and redox system. Metabolomics has made some progress in the study of plant biostress. Studies have shown that a class of low-molecular-weight lipid-soluble compounds with antimicrobial effects, including phenols, flavonoids, and terpenoids, produced by plants under stressful conditions or induced by excitons, are mainly produced and accumulate around the infestation site [1,6]. When maize is infested with anthracnose, terpenoid phytochemicals accumulate rapidly at the infestation site and surrounding tissues, enhancing disease resistance [7]. Hukkanen et al. demonstrated that the treatment of strawberries with BTH induced the production and accumulation of phenolics in strawberry leaves and fruits. BTH treatment induces the production and accumulation of phenolic substances in strawberry leaves and fruits and significantly enhances the resistance of strawberries to powdery mildew [8]. Plants produce defense-related secondary metabolites and ROS in response to adverse environments. These compounds can directly inhibit pathogenic bacteria’s growth and reproduction, reinforce plant cell walls, induce phytochelatin accumulation, and trigger HRs and programmed cell death. The production and accumulation of ROS constitute one of the initial responses to plant disease resistance, playing a crucial role in the defense mechanism against pathogens [9]. Gozzo et al. [8] showed that pathogenic bacteria infecting plants induce a burst of ROS in the host, which can cause the host to produce HRs. Currently, the main protective enzymes against plant disease include phenylalanine ammonia lyase, SOD, POD, CAT, PPO, and POX. PPO, chitinase, and β-1, 3-glucanas are involved in a variety of physiological and biochemical reactions, playing important roles in the induction of plant disease resistance. However, the early metabolite changes in maize after infestation with NCLB are still unclear.

This study was designed to explore the impacts of NCLB infection on the metabolic processes occurring in maize leaves during the silk emergence stage. By analyzing metabolic changes and identifying differential metabolites and pathways, the study sought to unravel the regulatory mechanisms involved in maize’s defense against NCLB. The findings provide theoretical and technical insights for early disease diagnosis, effective control strategies, and the development of resistant maize varieties.

## 2. Materials and Methods

### 2.1. Test Strains and Media

*Setosphaeria turcica* was provided by the Institute of Plant Protection, HAAS, Wuhan, China. It was inoculated in PDA medium and cultured in the dark at 25 °C. The PDA medium was prepared using 200 g of potatoes, 20 g of glucose, 20 g of agar, and 1 L of distilled water. Modified Fries medium was used for spore production and was formulated as follows: the composition of this medium included 30 g of sucrose, 0.5 g of yeast, 0.5 g of NaCl, 0.5 g of MgSO_4_, 0.1 g of CaCl_2_, 1 g of NH_4_NO_3_, 1 g of KH_2_PO_4_, 5 g of ammonium tartrate, and 1 L of distilled water.

### 2.2. Plant Material and Treatments

In 2022, the test was conducted at a research facility in Hulan District, Harbin City, located at an elevation of 130 m. The experimental region has an annual average temperature of 4.0 °C, with the temperature in the warmest month averages 23.1 °C. The yearly precipitation and annual average sunshine duration are approximately 505.4 mm and 2661.4 h, respectively. The typical frost-free period lasts 144 days. The maize variety used in this study was “Xianyu 335”, which is a nationally approved maize variety. Its female parent is PH6WC, and the male parent is PH4CV, both of which were bred by Pioneer Company. The seed germination rate was ≥90%. In the experimental area, the crop’s growth cycle was 127 days, accumulating temperatures exceeding 2700 °C.

Maize seeds were manually planted with precision, maintaining a row spacing of 70 cm, and the planting date was set for 10 May 2022. At the silking stage, the corn plants were inoculated with a spore suspension of NCLB. The thriving fungal blocks were washed with a 0.1% Tween 20 solution, filtered through two layers of gauze, and a spore suspension was prepared with a concentration of 1 × 10^5^–1 × 10^6^ spores/mL by using a hemocytometer. At the 11–12 leaf stage (big trumpet stage), spray inoculation was conducted on the surface of maize leaves, with the inoculation volume controlled at 5–10 mL per plant using clear water as the control. For each corn sample, we selected corn plants with uniform growth, chose a leaf from the same part of each plant, and collected six replicates for each treatment. Samples were promptly gathered, quickly frozen in liquid nitrogen, and stored at −80 °C until further processing for subsequent analyses.

### 2.3. Maize Leaf Metabolomic Analysis

After NCBL stress treatment, leaf samples from maize (tasseling stage) were collected from six plants (samples from plants growing without NCLB were used as the control). Samples were prepared from maize leaf samples using an extract solution (methanol/water = 3:1 with isotopically labeled internal standard mixture). LC-MS/MS analyses were performed using a UHPLC system with a UPLC HSS T3 column coupled to a Q Exactive HFX mass spectrometer. The raw data were converted to the mzXML format using ProteoWizard and processed with an in-house program. An in-house MS2 database (Biotree DB) was applied for metabolite annotation.

### 2.4. Statistical Analysis

An analysis of metabolite annotation was performed by PCA and OPLS-DA using SIMCA16 software. Student’s *t*-test was performed using SPSS 22 (VIP > 1.0 and *p* < 0.05). Changes in metabolites were indicated by the FC.

## 3. Results

### 3.1. Effect of NCBL on Physiological Performance of Corn

**Figure 1:**
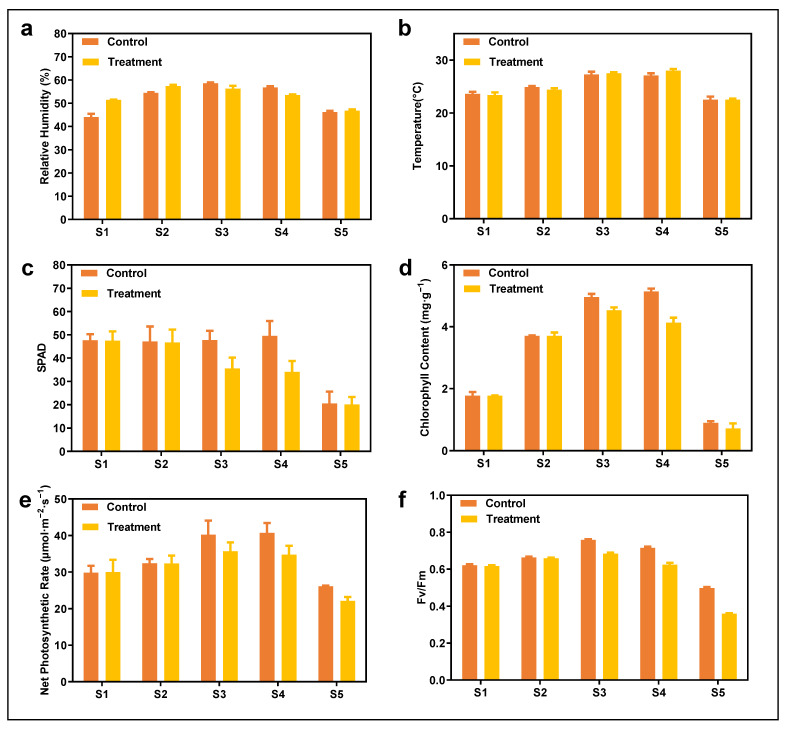
Changes in environmental factors and photosynthetic parameters at each stage of maize growth. (**a**) Relative humidity, (**b**) ambient temperature, (**c**) value of SPAD, (**d**) chlorophyll content, (**e**) net photosynthetic rate, (**f**) value of FV/FM. S1–S5, respectively, represent the seedling stage, elongation stage, tasseling stage, pustulation period, and mature period. At the silking stage, the corn plants were inoculated with a spore suspension of NCLB.

The effect of NCBL inoculation on growing maize is shown in Figure 1. The growth conditions for both the treatment and control groups were maintained consistently (Figure 1a,b), which is a fundamental requirement of experimental design to ensure the reliability of results. Prior to inoculation, the photosynthetic parameters of both groups were largely identical. Following infection by maize macrospots during the silking stage, the SPAD value of maize leaves decreased significantly. Compared with the elongation stage, the SPAD value declined by 11.08 (treatment). Relative to the control group, the SPAD value in the treatment group decreased from 12.132 to 35.568. These findings indicate that pathogen infection had a substantial impact on chlorophyll content in maize leaves. During the mature period, SPAD values for both the treatment and control groups reached their lowest points in the maize growth cycle, at 20.512 and 20.080 (Figure 1c), respectively. At the pustulation period, chlorophyll content in the control group peaked (Figure 1d). Changes in the net photosynthetic rate between the two groups both showed an initial increase followed by a decrease (Figure 1e). However, after NCLB infection, the rate of increase in the net photosynthetic rate in the treatment group was notably slower than in the control group, with a lower maximum value (35.71667 vs. 40.1833). Additionally, Fv/Fm values were measured for both groups, reaching their highest levels during the tasseling stage (Figure 1f). The Fv/Fm value for the control group was 0.074 higher than that of the treatment group.

### 3.2. Metabolomic Sample Quality Control Analysis

**Figure 2:**
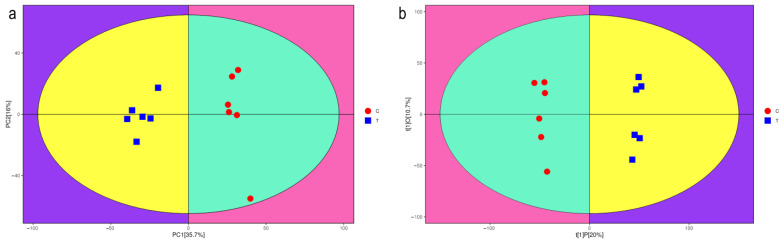
Score scatter plots of PCA and OPLS-DA models for group treatment (T) vs. control (C). (**a**) Score scatter plot of PCA model. (**b**) Score scatter plot of OPLS-DA model. Red dots represent the control group, while blue squares represent the treatment group. Each dot and square represents six repetitions of each treatment.

PCA clearly reflected the differences and similarities among treatments. To assess metabolomic changes following NCLB inoculation, we analyzed 17 organic acids, 12 amino acids, 10 sugars and derivatives, two alcohols, and seven other compounds. To further investigate the metabolic changes due to NCLB infestation, OPLS-DA was performed. The OPLS-DA score plot (b) showed that all samples conform to the 95% confidence interval of normal distribution with treatment groups well separated and no overlap. The OPLS-DA model explained 35.7% of the metabolite variability (*R*^2^ *X*). The overall goodness-of-fit (*R*^2^ *Y*) of the model was 0.994, and the overall cross-validation coefficient (Q2) was 0.869, indicating reliable results (Figure 2).

### 3.3. Differential Metabolite Analysis

#### 3.3.1. Holistic Analysis

**Figure 3:**
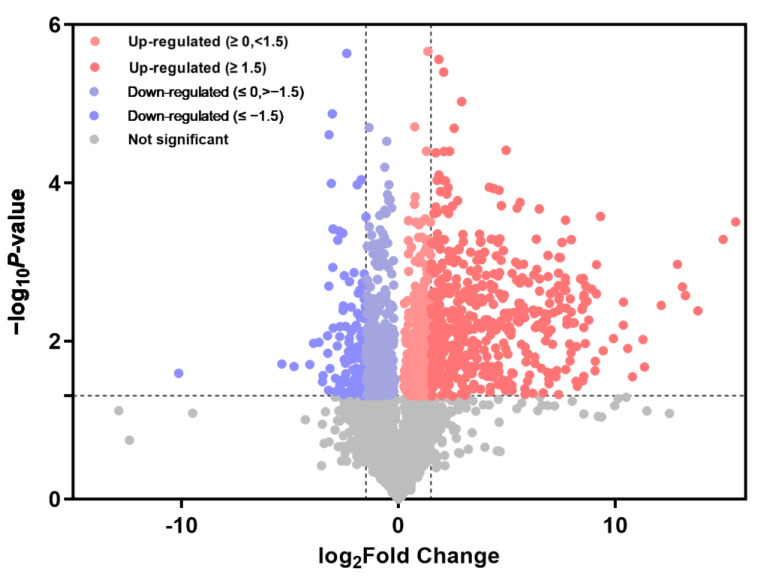
Volcanic diagram of differential metabolites under NCLB stress. Light-colored dots represent differentially expressed metabolites (DEM, Log2FC ≥ 0, <1.5 or Log2FC ≤ 0, >−1.5), while dark-colored dots represent significantly differentially expressed metabolites (DEM, Log2FC ≥ 0, <1.5 or Log2FC ≤ 0, >−1.5).

The volcano plot clearly shows the differences between metabolites in maize leaves in the treatment group (T) versus the control group (C) (Figure 3). Each point represents a metabolite, with the horizontal axis showing the fold change (taking the logarithm of the base of two) between groups and the vertical axis representing the *p*-value (taking the negative of the logarithm of the base of 10). The size of the scatter represents the VIP value from the OPLS-DA model, with larger points indicating higher VIP values. Point colors indicate metabolite significance, where red dots indicate significantly upregulated metabolites, blue dots indicate downregulated metabolites, and gray dots show no significant difference. Statistical analysis revealed 1032 upregulated and 242 downregulated metabolites following infestation.

#### 3.3.2. Qualitative and Quantitative Metabolite Analysis

Metabolites in maize plants infested with NCLB produce different responses, which can be analyzed using metabolomics to identify biologically and statistically significant differences. This helps elucidate the metabolic changes occurring in response to the disease.

**Figure 4:**
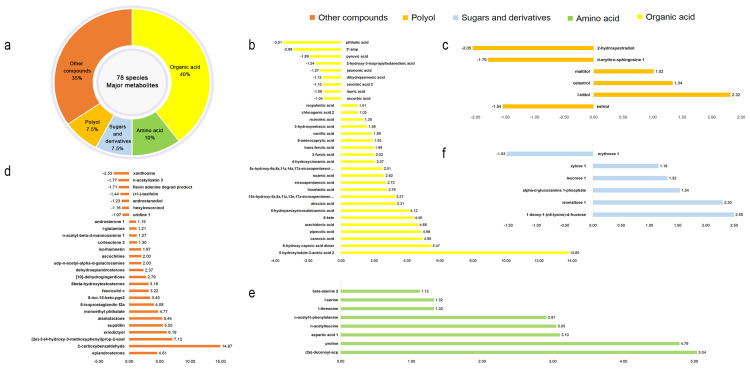
General diagram of quantitative analysis of differential metabolite Log_2_ FC values in maize leaves. (**a**) Classification of metabolites under S. turcica stress. (**b**) Log_2_ FC quantitative analysis of organic acids. (**c**) Log_2_ FC quantitative of polyols. (**d**) Log_2_ FC quantitative analysis of other compounds. (**e**) Log_2_ FC quantitative analysis of amino acids. (**f**) Log_2_ FC quantitative analysis of sugars and derivatives.

Maize leaf samples from both infected and uninfected groups were analyzed using GLC-MS. In this study, the value of variable importance in the projection (VIP) of the first principal component in OPLS-DA analysis was obtained. The metabolites with VIP > 1 and *p* < 0.05 (Student’s *t*-test) were considered as significantly changed metabolites. A total of 241 differential metabolites were identified. PCA and OPLS-DA were employed to assess the metabolite differences. Differential metabolites were selected based on Log_2_ FC < −1 and Log_2_ FC > 1, leading to the identification of 78 major metabolites (Appendix Table A1). These included organic acids (31, 40%), amino acids (8, 10%), sugars and derivatives (6, 7.5%), polyols (6, 7.5%), and other compounds (27, 35%) (Figure 4).

As shown in Figure 4, among the organic acids, levels of 5-hydroxyindole-3-acetic acid, 6-hydroxyhexanoic acid, and rhamnosus acid contents increased, with Log2 FC values of 13.80, 5.47, and 4.95, respectively. In contrast, phthalic acid, adenosine-3′-phosphate, and pyruvic acid contents declined, with Log_2_ FC values of −3.51, −2.89, and −1.89, respectively. Amino acids like cyclic leucine, proline, and aspartic acid showed marked increases (Log_2_ FC values of 5.04, 4.79, and 3.10, respectively). Among sugars and derivatives, only erythrose-1 showed a decrease (Log_2_ FC of −1.53), while other metabolites, including 1-deoxy-1 (n6-lysine)-d-fructose, isomaltulose-1, D-glucosamine-1-phosphate, trehalose, and xylose 1 increased, with log_2_ FC values of −1.53 each. Among polyols, the contents of L-aidulinol, southern serpentinol, and maltitol increased, with log_2_ FC values of 2.32, 1.34, and 1.02, respectively, while the contents of hydroxyestradiol, D-sphingosine, and estriol decreased, with log_2_ FC values of −2.05, −1.79, and −1.54, respectively. In the “other compounds” category, the contents of o-carboxybenzaldehyde, coniferaldehyde, and saccharinol increased, with log_2_ FC values of 14.97, 7.12, and 6.19, respectively, while xanthine nucleoside, 1-acetylisatin, and fluorine-ammonia anchorage decreased, with log_2_ FC values of −2.89, −2.53, and −1.71, respectively. Overall, there were notable differences in the amounts and contents of metabolites in the maize leaves under different treatments.

### 3.4. Differential Metabolite Metabolic Pathway Analysis

**Figure 5:**
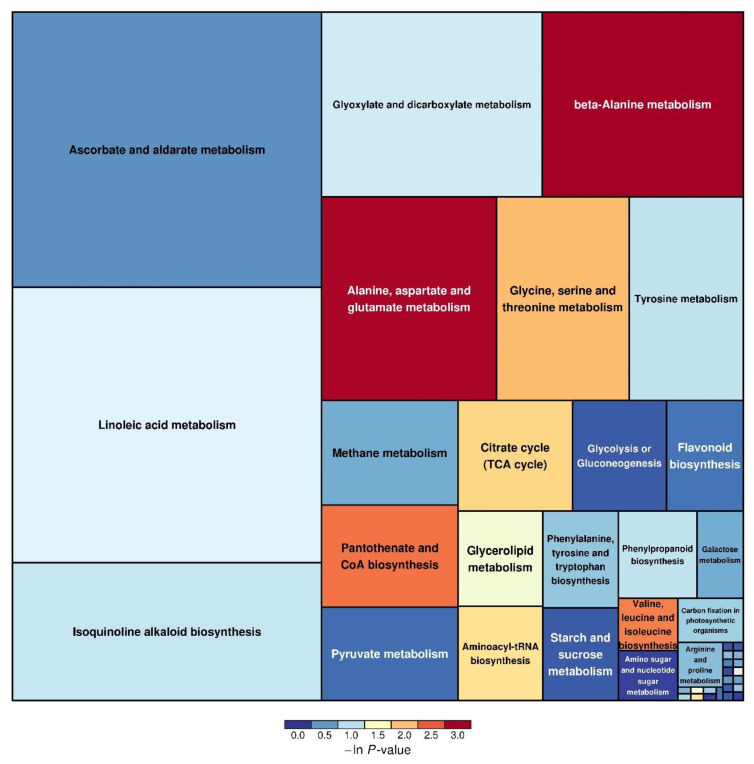
Metabolic pathway influencing factors. The size of the rectangle in the figure represents the size of the impact value; the larger the rectangle, the greater the impact. The warmth of the color indicates the size of the *p*-value, with colors closer to red signifying smaller *p*-values and greater significance in the enrichment analysis.

To more intuitively illustrate the variations in differential metabolites of maize leaves across different treatments, the metabolic pathway analysis results are presented using a rectangular graph (Figure 5). Each rectangle in the figure indicates a metabolic pathway. The horizontal coordinates and the extent of the rectangle represent the magnitude of the influence factors for that pathway in topological analysis. A larger size indicates a greater impact. The vertical coordinates of the rectangle and the color of the rectangle indicate the *p*-value of the enrichment analysis (taking the negative natural logarithm, i.e., ln(*p*)). The color gradient from cold to warm indicates the magnitude of the *p*-value, with red hues representing smaller *p*-values and higher significance in the degree of enrichment. As shown in Figure 4, the differential metabolites under NCLB infection were primarily enriched in the following metabolic pathways: ascorbic acid and aldehyde acid salt metabolism; linoleic acid metabolism; isoquinoline alkaloid biosynthesis; glyoxylate and dicarboxylic acid ester metabolism; beta-alanine metabolism; alanine, aspartate, and glutamate metabolism; glycine, serine, and threonine metabolism; tyrosine metabolism; methane metabolism; pantothenic acid and coenzyme A biosynthesis; and pyruvate metabolism.

### 3.5. KEGG Annotation of Differential Metabolites

**Figure 6:**
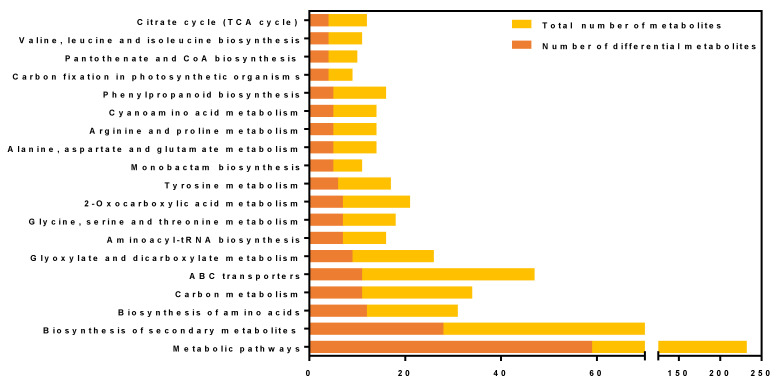
KEGG pathway annotation of differential metabolite in maize leaves. The yellow column represents the total number of metabolites, while the orange column represents the number of differential metabolites.

Based on the comparative analysis of the KEGG pathway database, it was found that differential metabolites were enriched in various metabolic pathways, as illustrated in Figure 6. The intricate metabolic reactions and their regulation within living organisms were accompanied by complex pathways and networks formed by different genes and proteins. Their interactions and mutual regulation ultimately result in systematic changes in the metabolome. Analyzing these metabolic and regulatory pathways offers a more comprehensive and systematic understanding of biological issues, including changes in biological processes due to variations in experimental conditions, mechanisms underlying traits or diseases, and the mechanisms of drug action. By utilizing gene and genome functional data, along with a focus on metabolic responses, we have linked potential metabolic pathways to their respective regulatory proteins, thereby elucidating the physiological and biochemical processes within cells. These processes encompass energy metabolism, material transport, signaling, cell cycle regulation, and information on conserved sub-pathways within the same lineage, which constitute the core of metabolic network research. Fifty pathways corresponding to the differential metabolite mapping of maize were compiled. The top five pathways were metabolic pathways, secondary metabolite biosynthesis, amino acid biosynthesis, carbon metabolism, and ABC transporters (refer to Appendix Table A2).

### 3.6. Analysis of Key Metabolic Pathways

Six phenolic synthesis pathways, such as those related to ascorbate and aldarate metabolism, linoleic acid metabolism, and isoquinoline alkaloid biosynthesis, undergo stress in plants following disease. In the ascorbate and aldarate metabolism pathway, one KEGG pathway was altered, with ascorbic acid (C00072) as the hit metabolite and an effect value of one. Similarly, in linoleic acid metabolism, one KEGG pathway was altered, with linoleic acid (C01595) as the hit metabolite and an effect value of one. For isoquinoline alkaloid biosynthesis, one KEGG pathway was altered, with L-tyrosine (C00082) as the hit metabolite and an effect value of 0.5. Two KEGG pathways were altered in glyoxylate and dicarboxylate metabolism, with isocitric acid (C00311) and citric acid (C00158) as the hit metabolites and an effect value of 0.47888. In beta-alanine metabolism, three KEGG pathways were altered, with beta-alanine (C00099), L-aspartic acid (C00049), and 1,3-diaminopropane (C00986) as the hit metabolites. Finally, in alanine, aspartate, and glutamate metabolism, four KEGG pathways were altered, with L-aspartic acid (C00049), succinic acid semialdehyde (C00232), L-glutamine (C00064), and pyruvic acid (C00022) as the hit metabolites (Table 1).

### 3.7. Chordal Analysis of Differential Metabolites

**Figure 7:**
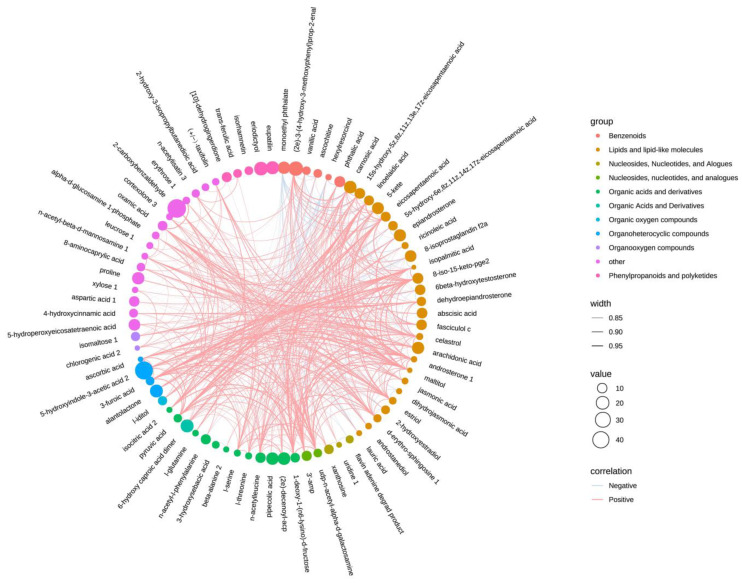
Chord plot analysis of differential metabolites in maize leaves. Spearman correlation between metabolites was calculated using R, with correlations whose absolute value was greater than 0.8. The group is represented by the supervised analysis. Correlation indicates positive or negative relationships, and the width of the edges represents the absolute value of the correlation. The size of the value corresponds to the log fold change.

Figure 7 presents a chord diagram constructed using the description and expression data of 78 differential metabolites, which were analyzed and grouped into 11 fractions. The majority of these metabolites showed positive correlations, while a few exhibited negative correlations or no correlation. Metabolites with larger dots were more strongly associated with others. These include pipecolic acid, 5-hydroxyindole-3-acetic acid 2, 6-hydroxy caproic acid dimer, (2e)-decenoyl-acp, alantolactone, (2e)-3-(4-hydroxy-3-methoxyphenyl)prop-2-enal, eriodictyol, and 2-carboxybenzaldehyde.

## 4. Discussion

Plant growth and development are frequently influenced by various biotic and abiotic stressors, prompting plants to activate defensive mechanisms to mitigate the effects of these stresses. To investigate the metabolic regulation mechanism of maize under NCLB infestation, this study detected 241 differential metabolites using GLC-MS. The differences in metabolite levels among the experimental sample groups were analyzed using PCA and OPLS-DA statistical methods. Log_2_ FC < −1 and Log_2_ FC > 1 were used as the criteria for screening differential metabolites, and 78 major differential metabolites were identified. These metabolites represent the most active compounds in maize leaves in response to NCLB stress and could potentially serve as biomarkers for maize defense against the fungus. Further metabolic pathway enrichment analysis revealed 50 altered metabolic pathways, including monobactam biosynthesis, carbon fixation in photosynthetic organisms, aminoacyl-tRNA biosynthesis, pantothenate and CoA biosynthesis, glycine, serine, and threonine metabolism, and amino acid biosynthesis. These pathways are all related to amino acid metabolism, organic acid metabolism, and biosynthesis. The TCA cycle, which is a key metabolic pathway for amino acid metabolism and the hub of various metabolic linkages and transformations [10], plays a crucial role in these processes. Under the stress of NCLB, maize experiences disruptions in homeostasis, requiring the synthesis of various substances to regulate osmotic pressure and physiological metabolism. These changes are essential for establishing a new homeostasis to maintain normal growth and development. These results suggest that maize leaves may alter the TCA cycle and phenylpropanoid metabolic pathways by increasing the content of relevant substances in amino and organic acid metabolism to improve tolerance to NCLB and reduce the extent of fungal attack.

### 4.1. Greater Metabolic Variation After Fungal Infection

In this study, we explored the metabolic response of maize leaves to infection by NCLB. The results from supervised OPLS-DA analysis indicated that all samples fell within 95% confidence intervals, with no overlap in distribution points and a clear separation between treatment groups. The OPLS-DA model explained 35.7% of the metabolite variability (*R*^2^ *X*). Among the 78 major differential metabolites identified, 58 were upregulated and 20 were downregulated, suggesting a significant metabolic response following maize leaf infection by NCLB. These differentially expressed metabolites were annotated to pathways such as alanine, aspartate, glutamate, and beta-alanine metabolism. Therefore, we speculate that some of the ultimate changes may be triggered by subtle differences between resistant and susceptible inbred lines during the early stages of fungal infection. Isoquinoline, as an alkaloid, exhibits notable antifungal properties and can effectively inhibit the synthesis by Fusarium acuminatum [11]. In a related study, Copley et al. integrated a transcriptional metabolomics approach to study primary metabolic regulation and antioxidant stress tolerance in soybeans after infestation with pathogenic fungi [12]. The results showed that there was a strong segregation of treatments based on the integration of the soybean leaf metabolome and transcriptome, and that strong fluctuating responses in glycolysis, the TCA cycle, and photosynthesis occurred in the primary metabolism of soybeans infested with *Rhizoctonia solani*.

### 4.2. Role and Regulation of Jasmonic Acid in Plant Defense Mechanisms

JA, as a plant hormone, produces a marked effect in the defense response of plants to biotic and abiotic stresses [13,14,15]. It enhances plant defenses against pathogens by modulating the immune response [16]. JA synthesis begins in plant *chloroplasts*, where it is synthesized, modified, and metabolized in peroxisomes, vesicles, and other organelles before being transported to the nucleus to exert its physiological effects [17,18]. JA enhances resistance to biotic stress by stimulating the degradation of the Jasmonate Zim-Domain protein and activating the downstream defense-initiating transcription factor MYC [19]. In the present study, JA in the differential metabolites of maize leaves infested with NCLB showed a decreasing trend, suggesting that the defense mechanism of maize leaves was disrupted by NCLB. Professor Liu Lijing’s team at the School of Life Sciences, Shandong University, found that plants enhance the immune response to Botrytis cinerea by inhibiting JA catabolism [20]. Kumaraswamy et al. reported that several metabolites are related to plant defense, including JA, dihydro-7-hydroxyglycine, glucoside, kaempferol-3-O-glucoside-7-O-rhamnoside, and methoxycinnamic acid (4-methoxycinnamicacid). Moreover, O-glucoside-7-O-rhamnoside and 4-methoxycinnamic acid were significantly increased [21]. In the α-linolenic acid metabolic pathway, linolenic acid is a direct substrate for JA synthesis [22]. Plant lipoxygenase activity can be enhanced by adding JAs, which promotes the synthesis of linolenic acid [23]. Additionally, 12-OPDA, an unsaturated fatty acid, is an intermediate in JA synthesis [24]. Enomoto found that ACOX and MFP2 are involved in the regulation of 12-OPDA synthesis during α-linolenic acid metabolism [25]. Exogenous MeJA regulates the expression of related genes to promote metabolite synthesis and improve the defense mechanisms of plants [26]. The above analysis suggests that signaling molecules in the α-linolenic acid metabolism and phytohormone signaling pathways are positively regulated by JAs, leading to the synthesis of metabolites and the adaptation of plants to stress.

### 4.3. Proline May Be Linked to Disease Resistance

Amino acids play a role as signaling molecules in plant disease resistance defense, and certain amino acids, such as lysine and isoleucine, can act as signaling molecules involved in disease resistance. Amino acids also play a role in plant resistance. For example, proline acts as a free radical scavenger that regulates plant cell redox homeostasis and participates in plant defense responses to biotic stress. Changes in amino acid levels can affect gene expression via transcription factors, thereby regulating plant disease resistance. In plants infected with pathogenic bacteria, proline levels increase significantly, suggesting that proline may be involved in plant defense responses against pathogens. For example, plant infection with Pseudomonas syringae significantly increases proline content in leaves [27]. In this study, amino acids were significantly elevated in the major differential metabolites of maize leaves infested and uninfested with NCLB, with a very high elevation in proline, suggesting that proline plays a positive role in NCLB resistance. Proline is an important osmoregulatory substance in plants’ responses to stress, and its increased content is often indicative of improved stress tolerance. Many studies have demonstrated that plants under stress show a rise in proline levels. For example, increased amino acid and organic acid content has been shown to enhance wheat’s stress tolerance [28,29]. Amino acid metabolism is related to plant osmoregulation and protein synthesis. After experiencing abiotic stress, plant cells stimulate amino acid metabolism to synthesize hydrophilic osmotic substances, regulate osmotic pressure, and maintain normal growth and development [30]. Free amino acids can directly or indirectly respond after stress [31], and aminoacyl-tRNA biosynthesis is an important pathway for protein formation [27]. Most of the amino acid differential metabolites in this study were upregulated by NCLB stress. This response may be due to the higher rate of protein catabolism compared to protein synthesis under stress conditions, which leads to an increase in amino acid levels.

### 4.4. Multifunctional Role of Sugar Metabolism in Resistance

Altered sugar metabolism is a hallmark of plant pathogens [32]. Alterations in sugar metabolism during plant–pathogen interactions play a key role in controlling the outcome of these interactions [33]. Both sugar-metabolizing enzymes and transport proteins from the host plant and pathogen are regulated to varying extents during these interactions [34]. Plant pathogens such as heterotrophs must draw sugars from the host plant to complete their life cycle, which triggers competition between plants and pathogens for sugar resources [35]. The regulation of the sugar supply between plants and pathogens is a key factor in determining disease resistance or susceptibility [36]. In this study, in comparison to uninfested NCLB maize leaf samples, the metabolite content of metabolites during the metabolism of NCLB-infested maize leaf samples was increased, with significant changes in sugars. Numerous bacterial toxins can enhance the permeability of plant cell membranes [37], facilitating the leakage of substances like sugars from within the cells [38]. When corn leaves are infected by the northern leaf blight pathogen, the cell membrane permeability may alter, resulting in the leakage of sugars from the cells and an elevation in sugar content within the leaf tissue. In addition, upon infection by pathogens, plants trigger a series of defense responses, including the activation of signaling pathways [39]. However, pathogens may disrupt the plant’s signal transduction system, impeding the plant’s ability to detect and respond to signals of pathogen infection. This, in turn, can affect the regulation and distribution of nutrients like sugars within the plant, ultimately leading to sugar accumulation in the leaves [40]. This suggests that sugars play a positive role in resistance to NCLB. β-1,3-glucan and chitosan are the main constituents of plant fungal pathogens, and together they form a reticulum in the cell wall [41]. Notably, most plants can be induced by excitons to produce and enrich β-1,3-glucan and chitinase in large quantities, which can effectively inhibit the growth of pathogens and degrade fungal cell walls in vitro, ultimately playing a role in the prevention of fungal diseases [42]. For example, Wang et al. showed that the activities of β-1,3-glucanase and chitinase activities in rice leaves improved the resistance of rice to rice blast [43].

### 4.5. Other Metabolites May Be Associated with Resistance

In this study, most of the other metabolites among the major differential metabolites in maize leaves, both infested and not infested with NCLB, showed an increasing trend. This suggests that these metabolites play a positive role in resistance to fungal infestation. Among these metabolites, five stand out for their significant role, namely 2-carboxybenzaldehyde, (2e)-3-(4-hydroxy-3-hydroxyphenyl)prop-2-enal, eriodictyol, eupatilin, and alantolactone. These metabolites may play a role in plant defense against fungal infections through their organic synthesis potential, chemical properties, and antifungal activity as volatile organic compounds [44,45]. 2-Carboxybenzaldehyde, for example, is a volatile organic compound that may be involved in plant interactions, including defense against diseases. Plant volatile organic compounds have potential applications in agricultural disease defense and control because they can act as natural antifungal compounds that inhibit the germination and growth of fungal spores [46]. Studies have shown that certain volatile aldehydes, such as trans-2-hexenal, have antifungal activity and can induce plant resistance to gray mold (caused by B. cinerea). Though the direct antifungal activity of 2-carboxybenzaldehyde has not been widely studied, similar aldehydes, such as trans-2-hexenal, are known for their antifungal properties, suggesting 2-carboxybenzaldehyde may have similar effects [47]. (2e)-3-(4-hydroxy-3-methoxyphenyl)prop-2-enal is a volatile organic compound (VOC) found in conifers that plays important roles in plant defense against fungal infections by exhibiting direct antifungal activity, promoting defense accumulation, and participating in plant interactions. Studies have shown that VOCs, such as pinene, inhibit fungal growth in conifers. These compounds help plants resist fungal infections by affecting their metabolism and growth. Eriodictyol produces a marked effect in plant defense against fungal infections by enhancing plant disease resistance through its antioxidant, anti-inflammatory, pathogen growth-inhibitory, and plant signaling pathway-modulating effects [48]. The ability of eriodictyol to inhibit the growth of rice blast fungus was also observed in a metabolomic analysis of rice varieties [49]. Eriodictyol was found to effectively protect against UV-induced keratinocyte death by inhibiting the cleavage of pro-caspase-3 or pro-caspase-9 and cytochrome C release, suggesting that it may provide protection to plant cells when they are attacked by pathogens. Eupatilin and alantolactone produce a marked effect in plant defense against fungal infections and in a variety of biological activities, including antifungal and anticellular growth. These effects help plants resist pathogens and protect plant health [50].

## 5. Conclusions

This study explored the effects of the stress caused by *Setosphaeria turcica* on the metabolic products and metabolic pathways of maize leaves through GC-MS technology combined with multivariate statistical methods. The results showed that although the NCLB did not cause irreversible damage to leaf growth, the contents of metabolites in related metabolic pathways had changed. The stress of the NCLB caused significant changes in the metabolites of maize leaves. Organic acids, amino acids, and sugars were the main differential metabolites, and their contents were mostly upregulated, indicating that organic acids and amino acid metabolites played a leading role in the resistance of maize leaves to the stress of the NCLB and maintained the normal physiological activities of the leaves together with other metabolites.

In addition, the content of phenolic compounds is higher under infection, and the related metabolic pathways for their synthesis may be activated to eliminate excessive ROS. The metabolic pathways of phenylpropanoid biosynthesis and flavonoid and flavonol biosynthesis are induced, and the expression of metabolites related to methyl jasmonate biosynthesis is upregulated. MeJA may alleviate the damage caused by the pathogen and enhance disease resistance by regulating metabolism. This study provides a theoretical basis for the in-depth exploration of the mechanism of maize plants’ tolerance to the NCLB. Future research can further explore the regulatory mechanisms and the role of key enzyme genes in metabolic pathways such as organic acids and amino acids in maize’s resistance to large spot disease stress, and it can explore the functions of phenolic compounds, methyl jasmonate, etc. Different maize varieties’ disease resistance mechanisms can be compared to screen for superior varieties or disease resistance genes. At the same time, the interaction between large spot disease stress and other environmental stresses can be studied, as well as the development of disease resistance technologies based on metabolic regulation, such as gene editing and metabolic engineering, to provide strong support for maize disease resistance breeding and production.

## Figures and Tables

**Table 1:** Key metabolic pathways.

Pathway Name	Number of Metabolites in the Pathway	Number of Hit Metabolites	*p*-Value	−log_10_(*p*)	Impact Value	Hit Metabolite Name and KEGG ID
Ascorbate and aldarate metabolism	14	1	0.63245	0.45815	1	Ascorbic acid (C00072)
Linoleic acid metabolism	5	1	0.29952	1.2056	1	Linoleic acid (C01595)
Isoquinoline alkaloid biosynthesis	6	1	0.34779	1.0562	0.5	L-Tyrosine (C00082)
Glyoxylate and dicarboxylate metabolism	17	2	0.32746	1.1164	0.47888	Isocitric acid (C00311); citric acid (C00158)
beta-Alanine metabolism	12	3	0.043571	3.1334	0.4359	beta-Alanine (C00099); L-aspartic acid (C00049); 1,3-diaminopropane (C00986)
Alanine, aspartate, and glutamate metabolism	21	4	0.050149	2.9928	0.41781	L-Aspartic acid (C00049); succinic acid semialdehyde (C00232); L-Glutamine (C00064); pyruvic acid (C00022)

## Data Availability

The original contributions presented in the study are included in the article, further inquiries can be directed to the corresponding author/s.

## Abbreviations

The following abbreviations are used in this manuscript:

NCLB	Northern corn leaf blight
PCA	Principal component analysis
OPLS-DA	Orthogonal partial least squares discriminant analysis
BTH	Benzothiadiazole
ROS	Reactive oxygen species
HRs	Hypersensitive responses
SOD	Superoxide dismutase
POD	Peroxidase
CAT	Catalase
β-1,3-glucanas	Β-1,3-glucanase
POX	Polyphenol oxidase
PDA	Potato dextrose agar
FC	Fold change
JA	Jasmonic acid
MeJA	Methyl jasmonate
HAAS	Heilongjiang Academy of Agricultural Sciences

## Appendix A

**Table A1:** Qualitative and quantitative analysis of differential metabolites in maize leaves.

Metabolite Type	Compound Name	Mean T	Mean C	VIP	Log_Fold Change
Organic acid	Carnosic Acid	2.11 × 10^−5^	6.80 × 10^−7^	2.14	4.95
	15s-Hydroxy-5z,8z,11z,13e,17z-Eicosapentaenoic Acid	2.27 × 10^−5^	2.35 × 10^−6^	1.98	3.27
	Linoelaidic Acid	2.23 × 10^−5^	3.21 × 10^−6^	1.97	2.79
	5-Kete	8.00 × 10^−5^	3.78 × 10^−6^	2.13	4.4
	Eicosapentaenoic Acid	6.11 × 10^−5^	9.19 × 10^−6^	2.09	2.73
	5s-Hydroxy-6e,8z,11z,14z,17z-Eicosapentaenoic Acid	2.66 × 10^−5^	4.68 × 10^−6^	1.78	2.51
	5-Hydroperoxyeicosatetraenoic Acid	1.59 × 10^−6^	9.00 × 10^−8^	1.89	4.12
	Ricinoleic Acid	5.66 × 10^−6^	2.22 × 10^−6^	1.76	1.35
	Jasmonic Acid	4.82 × 10^−4^	1.17 × 10^−3^	1.62	−1.27
	Isopalmitic Acid	7.00 × 10^−6^	3.48 × 10^−6^	1.65	1.01
	Dihydrojasmonic Acid	5.67 × 10^−5^	1.23 × 10^−4^	1.5	−1.12
	Trans-Ferulic Acid	1.47 × 10^−5^	3.72 × 10^−6^	1.89	1.98
	Pyruvic Acid	1.38 × 10^−3^	5.13 × 10^−3^	1.94	−1.89
	Ascorbic Acid	6.99 × 10^−6^	1.43 × 10^−5^	1.38	−1.04
	4-Hydroxycinnamic Acid	4.76 × 10^−6^	1.13 × 10^−6^	1.92	2.07
	Pipecolic Acid	2.12 × 10^−4^	7.19 × 10^−6^	2.19	4.88
	3-Furoic Acid	2.41 × 10^−5^	5.95 × 10^−6^	1.95	2.02
	Vanillic Acid	3.66 × 10^−6^	9.90 × 10^−7^	1.89	1.89
	8-Aminocaprylic Acid	1.36 × 10^−3^	3.59 × 10^−4^	1.96	1.93
	Abscisic Acid	2.30 × 10^−6^	2.30 × 10^−7^	1.76	3.31
	3-Hydroxysebacic Acid	3.43 × 10^−6^	1.15 × 10^−6^	1.46	1.58
	Chlorogenic Acid 2	8.00 × 10^−4^	3.93 × 10^−4^	1.49	1.03
	Phthalic Acid	3.56 × 10^−6^	4.04 × 10^−5^	1.34	−3.51
	Oxamic Acid	7.68 × 10^−5^	1.25 × 10^−5^	1.2	2.62
	Arachidonic Acid	8.73 × 10^−5^	3.41 × 10^−6^	2.11	4.68
	Lauric Acid	8.26 × 10^−5^	1.74 × 10^−4^	1.6	−1.08
	2-Hydroxy-3-Isopropylbutanedioic Acid	1.83 × 10^−5^	5.33 × 10^−5^	1.35	−1.54
	Isocitric Acid 2	4.84 × 10^−5^	1.04 × 10^−4^	1.85	−1.1
	5-Hydroxyindole-3-Acetic Acid 2	7.27 × 10^−5^	1.00 × 10^−8^	2.23	13.8
	3′-Amp	1.60 × 10^−7^	1.17 × 10^−6^	1.77	−2.89
	6-Hydroxy Caproic Acid Dimer	8.22× 10^−5^	1.85× 10^−6^	1.87	5.47
Amino acid	(2e)-Decenoyl-Acp	1.73 × 10^−4^	5.28 × 10^−6^	2.19	5.04
	Aspartic Acid 1	5.15 × 10^−3^	6.00 × 10^−4^	2.06	3.1
	N-Acetylleucine	2.01 × 10^−5^	2.43 × 10^−6^	2.05	3.05
	L-Threonine	4.48 × 10^−5^	1.80 × 10^−5^	1.78	1.32
	Proline	4.34 × 10^−4^	1.57 × 10^−5^	1.43	4.79
	L-Serine	4.06 × 10^−5^	1.62 × 10^−5^	1.73	1.32
	N-Acetyl-L-Phenylalanine	2.24 × 10^−6^	2.98 × 10^−7^	1.6	2.91
	Beta-Alanine 2	1.00 × 10^−4^	4.61 × 10^−5^	1.48	1.12
Sugars and derivatives	1-Deoxy-1-(N6-Lysino)-D-Fructose	9.19 × 10^−5^	1.63 × 10^−5^	2.02	2.5
	Xylose 1	1.09 × 10^−3^	4.89 × 10^−4^	2.05	1.16
	Leucrose 1	5.36 × 10^−5^	2.15 × 10^−5^	1	1.32
	Isomaltose 1	5.03 × 10^−4^	1.02 × 10^−4^	1.36	2.3
	Erythrose 1	2.18 × 10^−4^	6.28 × 10^−4^	1.21	−1.53
	Alpha-D-Glucosamine 1-Phosphate	5.59 × 10^−5^	1.92 × 10^−5^	2.04	1.54
Polyol	Estriol	7.13 × 10^−6^	2.08 × 10^−5^	1.59	−1.54
	L-Iditol	1.94 × 10^−4^	3.89 × 10^−5^	2.01	2.32
	2-Hydroxyestradiol	2.18 × 10^−6^	9.02 × 10^−6^	1.78	−2.05
	D-Erythro-Sphingosine 1	4.76 × 10^−5^	1.64 × 10^−4^	1.33	−1.79
	Celastrol	1.02 × 10^−5^	4.04 × 10^−6^	1.41	1.34
	Maltitol	2.38 × 10^−5^	1.17 × 10^−5^	1.56	1.02
Other compounds	Epiandrosterone	7.25 × 10^−6^	3.00 × 10^−7^	1.86	4.61
	6beta-Hydroxytestosterone	5.36 × 10^−6^	5.90 × 10^−7^	1.76	3.18
	Dehydroepiandrosterone	5.94 × 10^−6^	1.15 × 10^−6^	1.67	2.37
	[10]-Dehydrogingerdione	1.12 × 10^−6^	1.60 × 10^−7^	1.66	2.79
	Androstanediol	1.63 × 10^−4^	3.83 × 10^−4^	1.8	−1.23
	Cortexolone 3	2.89 × 10^−3^	1.18 × 10^−3^	1.68	1.3
	Androsterone 1	3.51 × 10^−4^	1.58 × 10^−4^	1.47	1.15
	Alantolactone	1.59 × 10^−4^	3.66 × 10^−6^	2.18	5.44
	8-Isoprostaglandin F2a	1.44 × 10^−6^	9.00 × 10^−8^	1.9	4.08
	8-Iso-15-Keto-Pge2	1.47 × 10^−6^	1.30 × 10^−7^	1.64	3.45
	Monoethyl Phthalate	1.19 × 10^−4^	4.37 × 10^−6^	2.02	4.77
	Udp-N-Acetyl-Alpha-D-Galactosamine	1.20 × 10^−6^	3.00 × 10^−7^	1.88	2
	(2e)-3-(4-Hydroxy-3-Methoxyphenyl)Prop-2-Enal	3.26 × 10^−5^	2.30 × 10^−7^	1.97	7.12
	Isorhamnetin	4.20 × 10^−6^	1.07 × 10^−6^	1.93	1.97
	Eriodictyol	2.51 × 10^−6^	3.00 × 10^−8^	1.99	6.19
	Fasciculol C	6.50 × 10^−7^	7.00 × 10^−8^	1.79	3.22
	N-Acetyl-Beta-D-Mannosamine 1	5.14 × 10^−4^	2.13 × 10^−4^	1.83	1.27
	Hexylresorcinol	1.15 × 10^−6^	2.57 × 10^−6^	1.53	−1.16
	Ascochitine	1.35 × 10^−5^	3.38 × 10^−6^	1.9	2
	Flavin Adenine Degrad Product	6.03 × 10^−5^	1.98 × 10^−4^	1.61	−1.71
	Eupatilin	2.57 × 10^−6^	5.00 × 10^−8^	2.03	5.55
	L-Glutamine	3.58 × 10^−5^	1.54 × 10^−5^	1.85	1.21
	Uridine 1	6.85 × 10^−5^	1.44 × 10^−4^	1.75	−1.07
	N-Acetylisatin 3	1.50 × 10^−5^	5.10 × 10^−5^	1.65	−1.77
	(+/−)-Taxifolin	9.50 × 10^−6^	2.57 × 10^−5^	1.14	−1.44
	2-Carboxybenzaldehyde	1.63 × 10^−4^	1.00 × 10^−8^	2.23	14.97
	Xanthosine	2.12 × 10^−6^	1.23 × 10^−5^	1.21	−2.53

**Table A2:** KEGG pathway annotation information.

Pathway	Description	Compounds (dem)	Compounds (all)	Percentage
zma01100	Metabolic pathways	59	173	34.10%
zma01110	Biosynthesis of secondary metabolites	28	76	36.84%
zma01230	Biosynthesis of amino acids	12	19	63.16%
zma01200	Carbon metabolism	11	23	47.83%
zma02010	ABC transporters	11	36	30.56%
zma00630	Glyoxylate and dicarboxylate metabolism	9	17	52.94%
zma00970	Aminoacyl-tRNA biosynthesis	7	9	77.78%
zma00260	Glycine, serine, and threonine metabolism	7	11	63.64%
zma01210	2-Oxocarboxylic acid metabolism	7	14	50.00%
zma00350	Tyrosine metabolism	6	11	54.55%
zma00261	Monobactam biosynthesis	5	6	83.33%
zma00250	Alanine, aspartate, and glutamate metabolism	5	9	55.56%
zma00330	Arginine and proline metabolism	5	9	55.56%
zma00460	Cyanoamino acid metabolism	5	9	55.56%
zma00940	Phenylpropanoid biosynthesis	5	11	45.45%
zma00710	Carbon fixation in photosynthetic organisms	4	5	80.00%
zma00770	Pantothenate and CoA biosynthesis	4	6	66.67%
zma00290	Valine, leucine, and isoleucine biosynthesis	4	7	57.14%
zma00020	Citrate cycle (TCA cycle)	4	8	50.00%
